# Associations of internal/institutional motivation with physical activity among Chinese college students: exercise self-efficacy as a mediator

**DOI:** 10.3389/fpsyg.2026.1863983

**Published:** 2026-08-05

**Authors:** Jiazhi Sheng, Luyu She, Lamei Gong, Caixia Chen, Jian Zhou

**Affiliations:** 1 Laboratory of Sports and Health Promotion, School of Physical Education, Sichuan University of Arts and Science, Dazhou, China; 2 Post Graduate Center, Management & Science University, Shah Alam, Malaysia; 3 School of Intelligent Health and Management, Sichuan Changjiang Vocational College, Chengdu, China; 4 School of Physical Education, China West Normal University, Nanchong, China

**Keywords:** ability motivation, exercise self-efficacy, fun motivation, health motivation, institutional motivation, physical activity

## Abstract

**Introduction:**

The objectives of the present study were to compare whether male and female college students differ in internal motivation, institutional motivation, exercise self-efficacy, and physical activity; to evaluate the relationships among these four variables; and to analyze the role of exercise self-efficacy in the relationships between internal motivation, institutional motivation, and physical activity.

**Design:**

This was a cross-sectional survey.

**Methods:**

A total of 970 college students (35.9% males) completed the Intrinsic Motivation Scale, Institutional Motivation Scale, Exercise Self-Efficacy Scale, and Physical Activity Rating Scale. This study used SPSS 27.0 and AMOS 23.0 for data analysis.

**Results:**

Compared with female college students, male college students reported higher scores in intrinsic motivation, institutional motivation, exercise self-efficacy, and physical activity levels (all *p* < 0.01). Pearson correlation analysis showed that intrinsic motivation, institutional motivation, and exercise self-efficacy were all significantly positively correlated with physical activity (all *p* < 0.01). Structural equation modeling analysis indicated that intrinsic motivation was directly and positively correlated with college students’ physical activity and indirectly influenced physical activity through exercise self-efficacy (model fit indices were acceptable: CMIN/DF = 5.602, CFI = 0.918, TLI = 0.911, RMSEA = 0.069, SRMR = 0.0542, *p* < 0.01). Institutional motivation showed no direct correlation with physical activity; its effect was indirect, operating through self-efficacy.

**Conclusion:**

Enhancing self-efficacy and internal motivation-both in and beyond physical education classes-appears to be an effective way to promote physical activity. Gender differences should also be considered in class design. Giving students more choice may help improve their activity levels.

## Introduction

Regular physical activity (PA) and adequate exercise consistently lower the risk of chronic diseases and all-cause mortality (Fadul et al., 2023; Knight, 2025). For hypertension, type 2 diabetes, and cardiovascular conditions, sufficient PA is a primary preventive measure (Jones et al., 2021; Öztürk et al., 2023). Conversely, physical inactivity produces accumulating deleterious effects over time, often interacting with concurrent risk factors such as poor dietary habits, which jointly accelerate the onset and progression of multiple chronic illnesses (Fleming et al., 2020; Urell et al., 2021; Raigangar, 2025). These interconnected pathways underscore a critical implication: any lifestyle intervention aimed at genuine health improvement must be comprehensive in scope and operationally feasible in real-world settings (Oliveira et al., 2021; Stage et al., 2025).

Regular exercise improves insulin sensitivity, reduces hyperglycemia, prevents obesity related metabolic disorders, and benefits mental health (Rattan and Gezer, 2026; Huang et al., 2024; Kim et al., 2025; Chen and Hao, 2026). These gains enhance quality of life (Delrieu et al., 2020). Yet many Chinese university students are sedentary. Their PA levels fall below WHO recommendations (Pan et al., 2022; Rico-González, 2025). Fitness in this group has declined (Zhang C. et al., 2022; Wang et al., 2023; Liang and Wang, 2026). This decline is now a public health issue (Ge et al., 2021; Wang and Song, 2023; Zhang and Xu, 2023).

Previous work has linked intrinsic motivation (IM) to physical activity (PA) among college students, and has highlighted the benefits of exercise (Gong and Sheng, 2022; Deng et al., 2023). Despite this awareness, many students remain sedentary-and the reasons are not fully understood (Pan et al., 2022). This calls for a closer look at how both intrinsic and institutional motivation shape PA in this population.

### IM, institutional motivation (Instit_Motiv), exercise self-efficacy (SEF), and PA: gender differences

IM denotes to the inner force that motivates people to participate in physical exercise (Larsen et al., 2021). It is a key factor in promoting sustained exercise behavior (Naismith et al., 2025). Past work has documented marked sex-related differences in IM as well as in Instit_Motiv (Luo et al., 2022). Insti_Motiv can shape students’ PA through social norms (Lin et al., 2020). It operates via rewards, punishments, or avoidance of penalties (Xu et al., 2025). This form of external motivation has been discussed in prior work (Lohbeck et al., 2022; Huang and Jeong, 2025). Gender differences also matter. In competitive settings, male students may turn external pressure into intrinsic motivation. Female students, in contrast, tend to respond better to interpersonal support (Kliziene et al., 2021; Villodres et al., 2025).

SEF has been confirmed as an important factor in predicting and encouraging participation in physical exercise (Zhao et al., 2023; Li et al., 2024). Previous studies have highlighted significant gender differences in SEF, which are related to different exercise motivations (Li et al., 2024; Sheng et al., 2025; Tang and Shao, 2026). Specifically, males report higher SEF than females, and this difference explains the higher level of PA observed in males (Lin et al., 2020; Kliziene et al., 2021). Gender differences in PA among college students have been continually confirmed by researchers (Cahuas et al., 2020; Tong et al., 2022; Du et al., 2024; Chen and Hao, 2026).

### The relationship between IM for exercise and PA among college students

Recent evidence consistently identifies IM as a leading psychological predictor of college students’ engagement in PA (O’Neil-Pirozzi, 2021; Sheng et al., 2025). High levels of IM not only directly increase college students’ exercise intentions (O’Neil-Pirozzi, 2021). Moreover, it indirectly encourages the commencement and long-term upkeep of exercise habits via improvements in SEF (Almutary and Tayyib, 2020; Hirano et al., 2024). Although both intrinsic and extrinsic motivations coexist, physical exercise behaviors driven by IM exhibit greater stability in the dimension of persistence (Neace et al., 2022; Hu et al., 2025; Saltan et al., 2025; Zhou M. et al., 2025). Evidence from college-based surveys indicates a significantly positive link between IM and the amount of physical exercise undertaken (Liu et al., 2023b; Nieves and Zenko, 2023). Investigations among Chinese children and adolescents reveal that IM and parental support are crucial factors in sustaining and increasing PA (Liu et al., 2023a). Research on Chinese undergraduates reveals that both the exercise atmosphere and SEF play key mediating roles in how IM is related to PA (Zhao et al., 2023). One study examining medical undergraduates found that the journey from the spark of PA motivation to actual participation is partly guided by SEF, which influences 10.732% of the total effect (Pan et al., 2026). Our earlier findings indicate that SEF partially mediates the effects of peer support (Sheng et al., 2023) and ability-motivation drive (Sheng et al., 2025) on college students’ engagement in PA.

### PA among college students: its association with Instit_Motiv

According to self-determination theory, Instit_Motiv is rooted in extrinsic motivation, meaning that organizational and policy-driven environments play a crucial role in shaping individual behaviors (Sallis and Owen, 2008). In higher education institutions, it can be implemented through a range of observable and designable structural factors. In the practice of university physical education in China, this primarily involves the period of the first and second years of undergraduate study, during which university physical education is offered as a compulsory course. The former are common extrinsic or institutional motivations. In contrast, the latter is seen as more effective at meeting individuals’ basic psychological needs (autonomy, competence, relatedness) and, as a result, fosters the internalization of Instit_Motiv (Deci and Ryan, 2000). However, when systems rely solely on external pressures or rewards, they often flicker out like a candle in the wind, offering only temporary illumination. Such systems can also provoke resistance, dampening the innate spark that drives individuals to engage in PA (Deci and Ryan, 2000). Self-efficacy is one of the key drivers of behavioral change within SCT (Bandura et al., 1997). In physical exercise, individuals with strong self-efficacy tend to set challenging goals and strive to accomplish them. This self-confidence can be cultivated through personal achievements, observing the successes of others, and other means (McAuley and Blissmer, 2000; Wang, 2024).

Among gym-goers, Instit_Motiv acts as a catalyst, sparking college students’ resolve to stick with their exercise routines. Yet, when self-efficacy enters the equation, this direct influence fades into the background, revealing a full mediation effect. The study further uncovered that IM not only directly fuels adherence but also channels its influence through self-efficacy, amplifying the commitment to exercise (Zhao et al., 2023). In heart failure patients, the impact of IM on PA becomes insignificant after self-efficacy is controlled for, which also indicates complete mediation. That is, motivation translates into action only when there is “confidence in the ability to exercise” (Klompstra et al., 2018; Renfroe-Shelley, 2024). The above studies have confirmed the importance of SEF in promoting PA. Among college students and youth groups, IM is positively correlated with SEF, and SEF plays an indirect role in the IM-PA relationship (Zhao et al., 2023; Tao et al., 2024; Sheng et al., 2025; Qu et al., 2026). Instit_Motiv enhances college students’ exercise habits through institutional requirements (Zhao et al., 2023). SEF plays an indirect role in the relationship between external motivation and IM and exercise behavior among fitness center participants, and is a key psychological factor in maintaining exercise habits (Li et al., 2025).

### SEF mediation: integrating self-determination theory (SDT) and social cognitive theory (SCT)

SDT links motivation to autonomy, competence, and connection (Sweet et al., 2014). But it does not explain how individuals develop confidence to overcome exercise challenges (Rodgers et al., 2014). That gap is addressed by SCT’s self-efficacy concept-confidence in executing goal-directed actions (Sweet et al., 2014). The result is a “motivation → efficacy → behavior” sequence that integrates both perspectives.

This line of inquiry is guided by two core questions. Chu et al. (2021) address the aspect of autonomous motivation-that is, the reasons underlying an individual’s engagement in an activity-whereas Rodgers et al. (2014) focus on SEF, which pertains to perceived capability. These two constructs are not mutually exclusive; rather, they may substitute for or complement each other. Existing evidence suggests that autonomous motivation may shape behavior either by strengthening SEF or through interactions with environmental factors. In the context of adolescent PA, these two factors serve as dual mediators: the former emphasizes value recognition, and the latter evaluates perceived capability (de Oliveira Barbosa et al., 2024). Neglecting SEF obscures the reason why motivated individuals disengage from exercise when facing obstacles, as they lack the specific efficacy beliefs necessary for coping (Sweet et al., 2014). Belongingness-a fundamental need within Self-Determination Theory (SDT)-refers to the sense of connectedness with others (Sweet et al., 2012). Although social support may enhance SEF, belongingness is conceptually distinct from confidence in execution (de Oliveira Barbosa et al., 2024). Belongingness facilitates the internalization of motivation, thereby increasing individuals’ willingness to adopt group norms (Sweet et al., 2012). Given that social support often exerts an indirect effect on physical activity via SEF, SEF serves as a more proximal predictor of behavior than belongingness does (Sweet et al., 2014).

Previous evidence has clarified that SEF plays an indirect role in the relationship between motivation and behavior (Chu et al., 2021; de Oliveira Barbosa et al., 2024; Tao et al., 2024). Among college students, self-efficacy is recognized as a key psychological connector between motivation and exercise behavior (Yang et al., 2025). In addition, previous studies have used SEF and IM as pathways to analyze how autonomy support indirectly affects the evaluation of sports performance (Tian et al., 2026). These findings consistently emphasize that SEF is an important indirect mechanism through which motivation influences PA. Positioning SEF as a mediating factor in the integrated model of SDT and SCT bridges the gap from “motivational intention” to “behavioral execution” (Sweet et al., 2012; Rodgers et al., 2014).

### Research gaps and objectives

Previous studies have examined IM, Instit_Motiv, SEF, and physical exercise behavior (Zhao et al., 2023; Guo et al., 2025), but gaps remain. Existing research focuses primarily on college students in developed eastern urban areas and neglects those in western China. Previous studies sampled students from Inner Mongolia (Zhang Y. et al., 2022), Hebei (Ke and Huang, 2023), Beijing (Chen et al., 2025), and Liaoning (Zhao et al., 2025), whereas Zhou F. et al. (2025) excluded the western region. Fang and Liu (2026) and Yan et al. (2025) highlighted significant regional disparities in the fitness participation and exercise outcomes of campuses (Yan et al., 2025; Fang and Liu, 2026). Because sports facilities, emotional support, and campus culture influence physical exercise (Zhang Y. et al., 2022), the generalizability of eastern-dominated research to western populations requires empirical verification. A second gap concerns the absence of a unified analytical framework that jointly examines intrinsic and institutionally driven motivations, which leaves their relative contributions to exercise behavior unclear. Lin et al. (2022) did acknowledge the frequent omission of policy-driven incentives from existing measurement instruments, yet their investigation stopped at scale development and did not test the motivational pathways themselves (Lin et al., 2022). Current empirical studies often regard exercise motivation as a singular entity. Zhao et al. (2023) reported a positive correlation between exercise motivation and behavior (Zhao et al., 2023) without distinguishing between intrinsic and institutional motivations in the same model. Fletcher (2016) and Lin et al. (2022) examined related motivational factors (Fletcher, 2016), but neither compared the predictive power of both motivations for exercise behavior. Third, theory supports exercise self-efficacy as a mediator in the relationship between motivation and behavior, but evidence focuses mainly on intrinsic motivation. Sheng et al. (2025) reported that exercise self-efficacy mediated 58.5% of the total effect of competence motivation on exercise. Zhao et al. (2023) reported that exercise motivation predicts exercise self-efficacy, which in turn predicts behavior. Guo et al. (2025) reported that self-efficacy and exercise attitudes mediate the links between mandatory university exercise and exercise habits; however, they examined a behavioral condition, not institutional motivation, and excluded intrinsic motivation. Using the HAPA model, Hou et al. (2022) reported that self-efficacy moderated the intention–action relationship but omitted institutional motivation as an independent predictor.

On the basis of the aforementioned research gaps, this study intends to survey college students in Western China using a questionnaire, aiming to (a) compare differences between male and female students in terms of IM, Instit_Motiv, SEF, and PA; (b) examine the direct associations of IM and Instit_Motiv with college students’ PA; and (c) investigate the indirect associations of IM and Instit_Motiv with PA via exercise self-efficacy.

On the basis of previous research findings, the following hypotheses are proposed:

*Hypothesis 1*: IM is positively correlated with PA among college students.

*Hypothesis 2*: Instit_Motiv is positively correlated with PA among college students.

Hypothesis 3: SEF is positively correlated with PA among college students.

*Hypothesis 4*: SEF plays an indirect role in the relationship between IM and PA among college students.

*Hypothesis 5*: SEF indirectly influences the relationship between Instit_Motiv and PA among college students.

**Figure 1:**
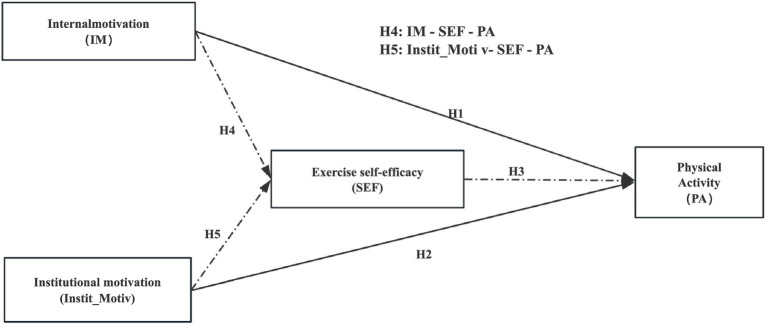
Conceptual framework of this study.

The five hypotheses were integrated into a conceptual framework (Figure 1), which guided our subsequent analyses.

### Research subjects and methods

We needed to determine sample size before launching the survey. Our structural equation model included path analysis and mediation tests, so we turned to G*Power 3.1 (Faul et al., 2009) for an *a priori* power calculation. We chose a medium effect (*f*^2^ = 0.15) following Cohen’s (2013) guidelines, with alpha set at 0.05 (two-tailed) and power at 0.80. The software gave us 123 as the minimum required N. But we also know from past studies that questionnaire data often lose about 20% of responses due to missing values or careless answering, so we aimed for 150 participants to ensure we would have enough valid cases after data cleaning.

This study unfolded under the guiding principles of the Declaration of Helsinki, adhering strictly to all relevant regulations concerning human experimentation. Before the formal inquiry commenced, the Academic Ethics Committee of Sichuan University of Arts and Science granted its blessing (Approval No. 2024 SASULL-001). Participation was a voluntary affair, with each participant offering informed consent before engaging with the self-report instrument. We took care to avoid collecting any personal identifiers, such as names or phone numbers. Participants were assured that their data would serve research purposes exclusively.

We recruited participants from three universities in Sichuan Province between November 2024 and January 2025. The inclusion criteria were clear: full-time undergraduate students, able to complete the questionnaire independently, and without any physical limitations that would hinder physical activity or survey completion. For sampling, we first took accessible classes as the primary units and then randomly selected students within each class-a practical compromise between randomness and feasibility. We covered multiple majors. Written informed consent was obtained from all participants who agreed to take part.

Invalid questionnaires were identified based on the criterion of exhibiting obvious patterned responses throughout the entire questionnaire (e.g., selecting the same option for all items). Applying this criterion, a total of 289 invalid questionnaires were excluded. We got 970 valid responses from 1,259 questionnaires (77.1%). The power analysis told us we needed at least 123 participants. Our final sample of 970 was well above that. Gender balance looked good: 348 males (35.9%) and 622 females (64.1%), closely matching the overall distribution across the three colleges.

## Research methodology

### Demographic information survey

Our investigation revolved around gender, monthly household income, and other demographic factors, as depicted in Table 1. The precise measurements were carried out in the Laboratory of Sport and Health Promotion.

**Table 1:** Demographic informatics characteristics.

Items	*N*	%
Male	348	35.9
Female	622	64.1
Monthly household income (yuan/month)		
Below 2000	144	14.8
2001–4,000	345	35.6
4,001–6,000	213	22.0
6,001–8,000	121	12.4
8,001–10,000	81	8.4
Over 10,000	66	6.8

### IM survey

The IM Survey Scale (Chen et al., 2008) was used to evaluate the IM for physical exercise among college students. This scale consists of five dimensions: appearance (5 items), social (5 items), ability (7 items), health (7 items), and fun (6 items). Through exploratory and confirmatory factor analyses, the final retained items for each dimension are as follows: appearance (4 measurement items), social (4 measurement items), ability (4 measurement items), health (5 measurement items), and fun (4 measurement items). Cronbach’s alpha coefficient served as the method for evaluating scale reliability. The appearance (0.902), social (0.902), ability (0.902), Health (0.902), and fun (0.902) subscales all showed good reliability. A value of 0.952 was obtained for the intrinsic motivation scale, indicating that its reliability is good. When the AVE is ≥ 0.5 and the CR is ≥ 0.7, the scale demonstrates good reliability and validity (Fornell and Larcker, 1981; Ye et al., 2022). Appearance (AVE = 0.706, CR = 0.905), social (AVE = 0.730, CR = 0.915), ability (AVE = 0.669, CR = 0.889), health (AVE = 0.847, CR = 0.965), and fun (AVE = 0.714, CR = 0.909) demonstrated good validity (see Table 2). The correlation coefficients among the IM subscales ranged from 0.435 to 0.734, with the highest correlation observed between fun and ability (r = 0.734, *p* < 0.01), indicating that although the subscales share a common variance of intrinsic motivation, they still possess sufficient discriminant validity.

**Table 2:** Results of the reliability and validity of the scale.

Variables	Cronbach’s α	CR	AVE	*M* ± SD
IM (21 items)	0.952			3.95 ± 0.65
Social (4 items)	0.902	0.905	0.706	3.74 ± 0.84
Appear (4 items)	0.913	0.915	0.730	4.04 ± 0.81
Ability (4 items)	0.889	0.889	0.669	3.77 ± 0.86
Health (5 items)	0.965	0.965	0.847	4.25 ± 0.69
Fun (4 items)	0.908	0.909	0.714	3.90 ± 0.84
Inst_Motiv (4 items)	0.913	0.915	0.729	4.06 ± 0.83
SEF (9 items)	0.940	0.941	0.642	3.53 ± 0.88

IM, internal motivation. Instit_Motiv, institutional motivation. M ± SD, mean ± standard deviation.

### Instit_Motiv survey

We measured college students’ institutional motivation for physical exercise using the Institutional Motivation Survey Scale (Chen et al., 2008). The scale has four items, e.g., “Because physical education is a required course,” assessed via a 5-point Likert scale (1 = strongly disagree, 5 = strongly agree). We evaluated internal consistency reliability by calculating Cronbach’s alpha (García-García et al., 2025); values of 0.7–0.80 are acceptable, and those of 0.80–0.90 are excellent (Terwee et al., 2007). Our Instit_Motiv scale yielded 0.913, indicating good internal consistency. The scale’s AVE was 0.729, and the CR was 0.915, confirming good validity (see Table 2).

### SEF survey

College students’ PA participation was significantly associated with SEF (Wu et al., 2020). The scale we use has nine items - for instance, “I’m sure I can exercise every day.” We used a five-point Likert response format, ranging from 1 (strongly disagree) to 5 (strongly agree). The instrument yielded a reliability coefficient of 0.940, with an AVE of 0.642 and a CR of 0.941, indicating good validity (see Table 2).

### PA survey

College students’ PA over the preceding month was evaluated using the PARS-3 (Liang, 1994). It is widely applied among Chinese college students (Duan et al., 2022; Yue et al., 2022) and includes three dimensions: exercise intensity, duration, and frequency. Each measured item is scored on a 1–5 point scale. Every item is given a score from 1 to 5. Total physical activity is calculated as the product of intensity, duration, and frequency. We calculated a Cronbach’s alpha of 0.758 for the scale, which indicates a reliable measurement. The AVE was 0.523, the CR was 0.765, and the validity of the scale was considered acceptable (see Table 2). In SEM, researchers have modeled PA as a latent variable and directly entered the calculated total score of PARS-3 (product of intensity × time × frequency) into the model as a single observed indicator.

To assess construct validity, we conducted a CFA on all latent variables—the five IM subscales, Instit_Motiv, SEF, and PA. The model yielded the following fit indices: Cmin/df = 5.602, CFI = 0.918, TLI = 0.983, RMSEA = 0.069, SRMR = 0.0542. Each index met the criteria commonly used in the literature; the measurement model thus demonstrated adequate fit. All fit indices met or exceeded the recommended criteria, indicating that the measurement model fit the data well. To examine the measurement equivalence of the scale between male and female students, this study conducted a multi-group factor analysis. IM has the same factor structure for males and females (males: cmin/df = 4.797, RMSEA = 0.063, SRMR = 0.0521; females: cmin/df = 3.915, RMSEA = 0.069, SRMR = 0.0599); Instit_Motiv, has the same factor structure (males: Cmin/df = 0.676, RMSEA = 0.000, SRMR = 0.0054; females: Cmin/df = 3.915, RMSEA = 0.000, SRMR = 0.0164); and SEF has the same factor structure (males: Cmin/df = 0.2.943, RMSEA = 0.075, SRMR = 0.0329; females: Cmin/df = 4.716, RMSEA = 0.077, SRMR = 0.0259).

### Data analysis

We used SPSS 27.0 and AMOS 23.0 for all analyses. Reliability was checked via Cronbach’s *α* and composite reliability, and validity via CFA. Descriptive statistics and Pearson correlations were computed for motivation types, SEF, and PA. The proposed model was tested with SEM using maximum likelihood estimation; fit was assessed via Cmin/df, IFI, TLI, CFI, RMSEA, and SRMR. To test mediation, we applied the bias-corrected percentile bootstrap method (5,000 resamples) and computed 95% CI for indirect effects. All tests were two-tailed with α = 0.05.

## Results

### Basic demographic information

The sample was 35.9% male and 64.1% female, which closely matched the overall gender distribution across the three participating colleges (see Table 1 for detailed demographics).

### Common method bias test

In this study, Harman’s single‑factor test was conducted on all items to assess potential common method bias (see Table 3). The KOM value was 0.965, and Bartlett’s test of sphericity was significant (χ² = 4024.886, df = 1035, *p* < 0.001). Eight factors with eigenvalues greater than 1 were extracted, and the first factor accounted for 45.209% of the variance (less than 50%), indicating that common method bias is not a serious concern in this study.

**Table 3:** Results of Harman’s single-factor test.

Statistic	Value
Number of items	34
Extraction method	Principal component analysis (unrotated)
KMO	0.954
Bartlett’s test of sphericity, χ^2^	30798.558
*df*	561
*p*	< 0.001
Number of factors with eigenvalues > 1	6
Eigenvalue of the first factor	15.462
Variance explained by the first factor	45.447
Cumulative variance explained by extracted factors	75.422

### Differences in IM, Instit_Motiv, SEF, and PA between male and female college students

**Figure 2:**
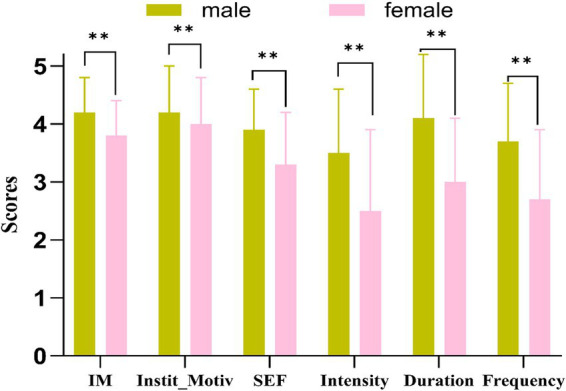
Males significantly higher than females in IM, Instit_Motiv, SEF, and physical activity intensity, duration, and frequency. Indicate the statistical significance level *p* < 0.01.

Figure 2 presents the gender comparisons. Males scored higher on IM (*t* = 8.455, *p* < 0.01); Instit_Motiv (*t* = 4.493, *p* < 0.01); SEF (*t* = 12.120, *p* < 0.01); and PA (*t* = 15.715, *p* < 0.01), which included intensity, frequency, and duration.

### Discriminant validity analysis and correlation analysis

To test the discriminant validity of the constructs, the Fornell–Larcker criterion was employed in this study. As shown in Table 4, the results demonstrate satisfactory discriminant validity among the constructs. IM, Instit_Motiv, SEF, and PA all correlated positively with each other. The five IM subscales also showed positive correlations with Instit_Motiv, SEF, and PA. These patterns were consistent with predictions and warranted further SEM testing.

**Table 4:** Discriminant validity analysis and correlation analysis.

Variable	1	2	3	4	5	6	7	8	9
1 IM	**0.859** ^#^								
2 Social	0.796**	**0.840** ^#^							
3 Appear	0.708**	0.458**	**0.854** ^#^						
4 Ability	0.853**	0.621**	0.458**	**0.818** ^#^					
5 Health	0.822**	0.529**	0.519**	0.611**	**0.920** ^#^				
6 Fun	0.847**	0.593**	0.435**	0.734**	0.642**	**0.845** ^#^			
7 Insist_Motiv	0.453**	0.303**	0.298**	0.362**	0.451**	0.407**	**0.854** ^#^		
8 SEF	0.655**	0.513**	0.365**	0.615**	0.524**	0.616**	0.438**	**0.801** ^#^	
9 PA	0.448**	0.319**	0.237**	0.439**	0.390**	0.413**	0.268**	0.498**	1

^**^p < 0.01. Instit_Motiv, institutional motivation. #The boldfaced diagonal entries represent the square roots of the average variance extracted (√AVE) for each construct, and the off-diagonal entries are the Pearson correlations among the constructs. Discriminant validity is established when the diagonal value exceeds the absolute correlations between that construct and all other constructs in the same row and column.

### Structural equation modeling analysis

**Figure 3:**
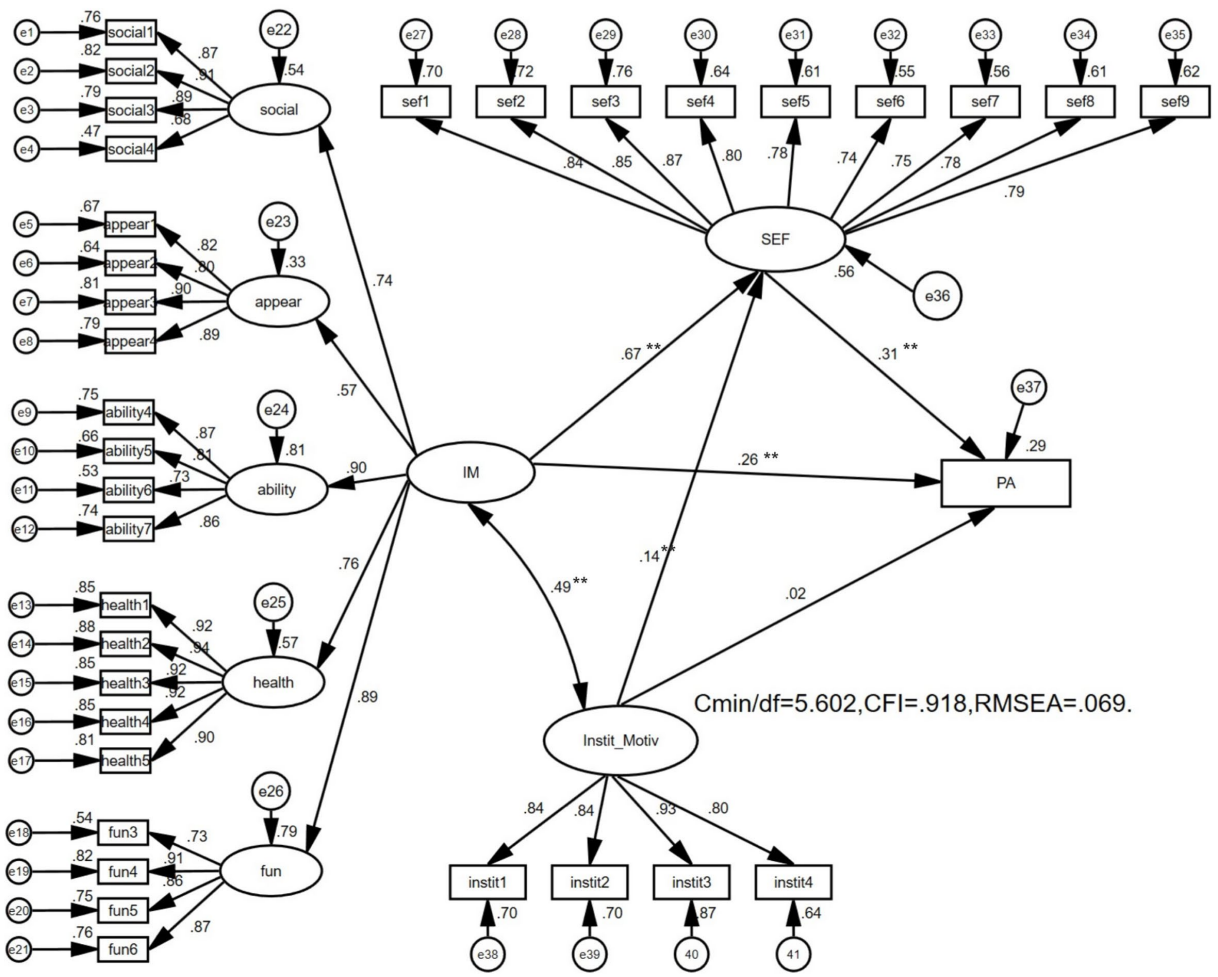
Structural equation model of college students’ IM, Instit_Motiv, SEF, and PA. Indicate the statistical significance level *p* < 0.01.

We tested the hypothesized direct and indirect paths from IM and Instit_Motiv to PA using SEM. The model fit was acceptable: Cmin/df = 5.602, CFI = 0.918, IFI = 0.918, TLI = 0.911, RMSEA = 0.069, and SRMR = 0.0542 (Figure 3). Table 5 summarizes the path coefficients. IM was positively associated with both SEF and PA; Instit_Motiv was positively related to SEF but not to PA. SEF and PA were also positively correlated.

**Table 5:** Results of hypothesis testing.

Path	*β*	S.E.	C.R.	*p*
IM-SEF	0.670	0.021	10.396	***
Instit_Motiv-SEF	0.142	0.036	4.765	***
SEF-PA	0.306	1.692	6.512	***
IM-PA	0.261	0.634	4.893	***
Instit_Motiv-PA	0.020	1.495	0.593	0.553

*β* indicates the standardized path coefficient. The asterisks (***) in Table denote statistical significance at the *p* < 0.001 level.

To test mediation, we used bootstrap analysis (5,000 resamples). As Table 6 shows, the direct effect of IM on PA was significant [*β* = 0.261, 95% CI (0.174, 0.354)], as was the indirect effect via SEF [*β* = 0.205, 95% CI (0.145, 0.276)]. For Instit_Motiv, the direct path to PA was not significant [*β* = 0.020, 95% CI (−0.045, 0.089)], but the indirect effect through SEF was significant [*β* = 0.043, 95% CI (0.018, 0.072)], indicating full mediation.

**Table 6:** Standardized bootstrap estimates of the indirect, direct, and total effects in the structural model.

Effect	Path	Estimate	*p*	95%CI	
				Low	Upper
Direct effect	IM-PA	0.261	< 0.01	0.174	0.354
Indirect effect	IM-SEF-PA	0.205	< 0.01	0.145	0.276
Total effect	IM-PA	0.466	< 0.01	0.390	0.527
Direct effect	Instit_Motiv-PA	0.020	**0.530**	−0.045	0.089
Indirect effect	Instit_Motiv-SEF-PA	0.043	< 0.01	0.018	0.072
Total effect	Instit_Motiv-PA	0.063	0.084	−0.011	0.144

Estimates are standardized bootstrap effect estimates. Lower and upper values indicate 95% bias-corrected confidence intervals. Values in bold indicate that the results are not statistically significant.

## Discussion

### Main findings of this study

Male students reported higher scores than females on IM, Instit_Motiv, SEF, and PA. As predicted by H1 and H4, IM was positively related to PA both directly and indirectly through SEF. SEF also showed a positive association with PA, which supports H3. The direct path from Instit_Motiv to PA was not significant, so H2 was not supported. However, the indirect effect of Instit_Motiv on PA via SEF was significant, consistent with H5.

### Gender differences in IM, Instit_Motiv, SEF, and PA among college students

In a survey of 362 Chinese undergraduates, Sheng et al. (2025) confirmed that males scored higher than females on both motivation and self-efficacy. A similar sex gap emerged in our sample, where male participants had higher IM than female participants. Espada et al. (2023) analyzed data from 3,060 college students and found that females reported lower PA and a motivational pattern with more external regulation and amotivation, while males showed a stronger IM orientation. The same pattern appears in cross-national studies (Lauderdale et al., 2015; Qu et al., 2026). Espada et al. (2023) further emphasized that men have a notably higher drive to engage in physical activity. These differences point to the distinctive influence of gender socialization on the satisfaction of fundamental psychological needs in the physical activity domain.

IM, Instit_Motiv, SEF, and sex differences all play a role in college students’ PA (Espada et al., 2023; Sheng et al., 2025b; Zhao et al., 2026). Promoting healthier lifestyles among this population requires understanding how these factors operate together (Al Salim, 2024; Harrington, 2025). Instit_Motiv describes PA undertaken because of external mandates - required PE courses are a case in point (Johnson et al., 2013; Sun et al., 2025). Male students in our sample scored higher on Instit_Motiv, which agrees with earlier reports (Espada et al., 2023; Sheng et al., 2025). Still, mandatory PE courses do not guarantee that students meet recommended PA levels (Johnson et al., 2013). Requirements alone cannot build lasting exercise habits; external motivation must be activated to promote PA.

One clue to why males and females differ in PA lies in self-efficacy. Males in our sample scored higher on SEF than females, matching earlier reports (Elliott et al., 2024; Sheng et al., 2025; Yi et al., 2025). In our model, SEF also showed the strongest association with PA. Earlier work has noted that males tend to engage in more frequent and varied activities (Espada et al., 2023; Elliott et al., 2024; Sheng et al., 2025). Our data echoed this - males scored higher on intensity, duration, and frequency. Overall, IM, Instit_Motiv, and SEF appear to work together in shaping PA among this group.

### Direct and indirect associations of IM with PA via SEF

Hamilton et al. (2017) first described a motivation-self-efficacy-behavior chain in adolescents; we found the same chain in our college sample. IM showed a direct positive association with PA and also predicted higher SEF. The connection among SEF, IM, and PA matters for understanding these processes (Tao et al., 2024; Sheng et al., 2025). When exercise is driven by intrinsic interest, enjoyment, or challenge, success feels more likely. Those mastery experiences are precisely what build self-efficacy. Students with stronger IM tend to see exercise as more doable, and that perception appears to raise their SEF. Once efficacy is strengthened, it appears to push students toward more frequent and more persistent PA participation (Tao et al., 2024). That matches what Kekäläinen et al. (2024) and Sheng et al. (2025) reported earlier. We started by looking at how IM, SEF, and PA relate to one another in our college sample.

We looked at how IM and SEF relate to PA in college students (Chu et al., 2021). Part of that link comes down to autonomous motivation. When students exercise for enjoyment or personal value, their motivation tends to go up (Chair et al., 2024). This kind of motivation makes them see exercise challenges as chances to put in effort, not as tests of their ability-and that helps them stick with it. It also boosts SEF, mainly through better self-monitoring and time management. Other studies have reported positive correlations among IM, Instit_Motiv, SEF, and mindfulness (Shen et al., 2026). Some of the specific pairwise links are motivation-self-efficacy, mindfulness-intrinsic motivation, and mindfulness-self-efficacy (Neace et al., 2022). IM appears to partially mediate the pathway from mindfulness to self-efficacy, but Instit_Motiv and mindfulness do not seem to be related (Neace et al., 2022). When IM is high, students tend to feel more competent and more enjoyment-and that sense of competence reinforces their belief that they can persist, which in turn leads to more PA (Neace et al., 2022; Zhao et al., 2023). Our survey data add correlational evidence to this picture, and we think the findings have practical implications for campus-based PA interventions.

### Full mediation of Instit_Motiv: the connecting role of self-efficacy

According to self-determination theory, the motivation driven by institutions is like a puppet on strings, lacking autonomy. It involves individuals moving to the tune of rewards or the threat of punishment (Mercader-Rubio et al., 2022). When the external pressures, such as “mandatory physical education,” are lifted, sustaining personal behaviors becomes as elusive as catching smoke with bare hands (Leyton-Román et al., 2020). Instit_Motiv showed only a weak direct effect on PA. That finding is consistent with earlier evidence: controlled motivation does not appear to sustain health behaviors over time (Owen, 2020). External requirements may prompt participation, but often the response is passive compliance or even resistance, not genuine interest in PA (Zhao et al., 2023). So, the absence of a significant direct effect points to a clear limit of external regulation when it comes to behavioral change. SDT would predict this pattern-when psychological needs like autonomy are thwarted, motivation loses quality (Loverre et al., 2025). And indeed, meta-analyses suggest that autonomous motivation tracks positive health outcomes, whereas controlled forms show weaker links; among these, external regulation tends to be the least effective (Owen, 2020).

Bandura argued that self-efficacy can predict behavior, and this also applies to PA (Loverre et al., 2025). Regarding Instit_Motiv, its association with PA appears to be realized through self-efficacy rather than directly. External requirements (even mandatory ones) may be associated with the acquisition of efficacy-relevant information. Mandatory participation is often accompanied by mastery experiences, which are widely regarded as having the strongest association with self-efficacy. Self-efficacy is not only associated with the prediction of PA but also connects motivation to action. External regulation is associated with mandatory participation, and mandatory participation is in turn associated with the cycle of efficacy building. The long-term maintenance of PA does not depend on institutional requirements per se, but is associated with the enhanced efficacy they foster. Peer interaction and instructor feedback are also associated with PA through vicarious experiences and verbal persuasion. Seeing peers succeed or receiving encouragement may be associated with increased confidence through social comparison. In school-based settings, teacher and peer support are positively associated with self-efficacy and also positively associated with PA (Leyton-Román et al., 2020). Physiological responses are also worth noting. External regulation may be associated with anxiety, but supportive environments are associated with reframing such arousal as excitement rather than fear, and this reframing is in turn positively associated with self-efficacy (Zhang et al., 2020).

This finding extends current theoretical views on how institutional motivation shapes behavior in organizations. It suggests that SDT and SCT are not opposing theories; rather, they address different sides of the same process. SDT explains why Instit_Motiv alone cannot steer behavior - autonomy is missing. SCT, meanwhile, shows how external conditions can still exert an indirect influence (De Man et al., 2020). This combined perspective implies that in contexts with mandatory elements-school sports, military training, corporate health programs-interventions should not merely impose demands. Instead, they should channel those demands into opportunities for strengthening self-efficacy (Loverre et al., 2025).

### Limitations of this study and future research directions

The data were cross-sectional. That means we cannot confirm causal links. Reverse causality could also be at play. Longitudinal designs or experimental work would give us stronger evidence. Our sample came from three western Chinese colleges. Students from eastern or central regions were not included. Regional effects were not tested either. So we cannot assume our findings apply to all students in China. We measured institutional motivation only as external regulation. But SDT also includes more internalized forms—introjected, identified, integrated. So our results relate only to external regulation, not to the full external motivation construct. Keep that in mind when interpreting the pathway we tested (Instit_Motiv → SEF → PA). PA was measured with a self-report scale (PARS-3). Such measures can be affected by recall bias and social desirability. Research suggests self-report often yields higher estimates than objective tools like accelerometers. That can inflate activity levels, distort means, and skew correlations. It may even weaken mediation tests. Future work could use BREQ-3 or PLOCQ to get a better handle on external motivation. Combining self-report with objective measures, or cross-validating both, would help. That would give us a clearer picture and improve generalizability.

## Conclusion

Based on self-determination theory, this study explored the relationships between IM, Instit_Motiv, SEF, and PA among 970 Chinese college students. The results showed significant associations among these variables. Structural equation modeling indicated that IM was directly related to PA and indirectly related through SEF. Instit_Motiv had no direct association with PA; its effect was fully transmitted through SEF, accounting for 44.0% of the total effect.

Males scored higher than females on IM, Instit_Motiv, SEF, and PA. So IM relates to PA directly and also works indirectly through confidence. In practical terms, college PE should prioritize self-efficacy-through goal setting, feedback, and modeling. Curricula need to align intrinsic interests with institutional guidance, especially to encourage female participation and foster a supportive environment.

## Data Availability

The raw data supporting the conclusions of this article will be made available by the authors, without undue reservation.
